# Freiburg mindfulness inventory (FMI) short form and revised form (FMI-13R) — norm scores and psychometrics in a representative German sample

**DOI:** 10.1186/s40359-025-03671-3

**Published:** 2025-11-26

**Authors:** Harald Walach, Sebastian Sauer, Niko Kohls, Nina Rose, Stefan Schmidt

**Affiliations:** 1 Next Society Institute, Kazimieras Simonavicius University, Vilnius, Lithuania; 2 Change Health Science Institute, Basel, Switzerland; 3 Dept. Psychology, University of Applied Sciences, Ansbach, Germany; 4 Faculty of Applied Natural Sciences and Health, Coburg University of Applied Sciences, Coburg, Germany; 5 Private practice, Oldenburg, Germany; 6 Dept. of Psychosomatic Medicine and Psychotherapy, Medical Faculty, Medical Center - University of Freiburg, University of Freiburg, Freiburg, Germany; 7 Institute for Frontier Areas of Psychology and Mental Health (IGPP), Freiburg, Germany

**Keywords:** Assessment of mindfulness, Acceptance, Presence, FMI, Norm data

## Abstract

**Background:**

The Freiburg Mindfulness Inventory (FMI) in its short form is one of the most frequently used instruments in research to measure self-attributed mindfulness levels. Despite the widespread use, there is no calibration sample available until now. Thus, we decided to calibrate it in a representative German sample as norm values are of relevance for clinicians and researchers alike. In addition, we tried to replicate the instruments’ psychometric properties.

**Methods:**

A sample of 1,012 respondents was recruited from a commercial online panel, approximating a representative sample of the German population. Participants filled in the FMI-14 (short from). We performed psychometric analyses and calculated a Confirmatory Factor Analysis (CFA). We also computed a measurement invariance analysis and a LASSO regression to identify population variables that predict FMI scores. To gauge external validity of the instrument, we also presented the FMI together with four items of the Patient Health Questionnaire (PHQ-4).

**Results:**

We obtained the best psychometric properties with a revised version of the FMI-13R, where the only negatively coded item was removed due to lack of fit. McDonald’s Omega was found to be 0.88 and the mean item-scale intercorrelation was 0.36. The construct is unidimensional, with two highly correlated subconstructs, Presence and Acceptance (*r* = 0.93), which can be separated for conceptual reasons if desired. The CFA supported these models. Age and gender stratified norm scores were calculated and reported. Regression analyses and partial correlation analyses showed negative correlation of FMI-13R scores with measures of anxiety and depression, as expected, and thereby support our previous findings that the instrument is valid and that the Acceptance component of mindfulness is the one that is associated with positive effects on health-related parameters.

**Conclusion:**

The FMI-13R is a psychometrically sound and valid instrument for the assessment of mindfulness. Norm scores can now be used by clinicians and researchers to classify individuals or samples into a range of values compared to the German population.

**Supplementary Information:**

The online version contains supplementary material available at 10.1186/s40359-025-03671-3.

## Background

Mindfulness research is well established; a Google Scholar query yielded 1,400,000 hits (May 2025). Mindfulness-based interventions have been found to be effective in reducing a plethora of symptoms related to various mental health conditions, including depression, anxiety, stress, and chronic pain [1–12]. Research suggests that mindfulness may help to improve various psychological conditions such as emotion regulation [13], attention and focus [14–16]. and overall well-being [17–19].

To operationalize the construct of mindfulness, various questionnaires have been developed that use self-attributions of behaviors and mental states [20, 21]. Although such a procedure is not foolproof and not entirely valid in all situations [22], it is still considered the most parsimonious and easiest way to assess mindfulness both as a state or a trait (see [23, 24] for behavioral approaches). Following a well-known tradition in psychometric research, one can operationalize mindfulness either as a transitory and changing state [25–27], or as a trait that is more stable [28–30].

The *Freiburg Mindfulness Inventory* (FMI; *Freiburger Fragebogen zur Achtsamkeit* – FFA) was one of the earliest instruments and has the advantage that its 30 items were developed through extensive background research of Buddhist texts and interviews with experts, including a feedback cycle [31–33]. An English translation was published soon after [34]. Using a larger validation sample, a short version was constructed [35], comprising 14 items. The long version contained items that caused comprehension problems among people without meditation experience. The short version, from which problematic items were removed, proved more robust and easier to understand for people without meditation experience, making it suitable for assessing self-attributed mindfulness in everyday contexts. Therefore, the present contribution focuses on the short form only, as it is the version predominantly used.

Depending on the framing question that instructs respondents to apply the questions to a specific time frame, the instrument can be used to assess changing states or more enduring traits. For instance, for assessing changes before and after a training course, one can contextualize the time frame for which the items are to be rated as “Please use <the last hour/ couple of hours/ day/ couple of days > as the time frame to consider each item”. If a more enduring trait is to be assessed, one can adapt the time frame to larger periods, such as “Please use the last <14,/ 30 days/…>as the time frame to consider each item”. The default setting for the time frame is 14 days.

A confirmatory factor analysis [36] replicating the original psychometric findings in an online convenience sample, revealed a general factor that is comprised of two highly correlated subfactors: *Presence* (six items) and *Acceptance* (eight items). Partial correlation analyses revealed that mindfulness is beneficially associated with lower depression and anxiety, and that this effect is driven mainly by the *Acceptance* component of mindfulness. Thus, the acceptance component seems the key mediator in the beneficial effects of mindfulness on reducing depression and anxiety, highlighting its significance in mindfulness-related health outcomes. This finding is backed up by similar conceptualizations of mindfulness in the literature (e.g [37, 38]). Furthermore, there is some evidence that one of the other frequently used mindfulness scales, the Five Facts Mindfulness Questionnaire (FFMQ), also conforms to such a temporal two-component model [39]. Consequently, with regard to convergent and divergent validity but also for practical reasons, this two-factor model is therefore particularly recommended for empirical research exploring the relationship between mindfulness and health-related outcomes.

The instrument has been well received and exists in a variety of languages. To our knowledge, the following translations or adaptations exist: French [40], Chinese [41], Italian [42], Portuguese [43, 44], Persian/Farsi [45], Spanish [46, 47], Dutch [48], Indonesian [49], Turkish [50], Polish [51, 52], Arabic [53], Korean [54] and Japanese [55]. All studies indicate that the translated FMIs demonstrate good psychometric properties, with high internal consistency and a comparable factor structure. Four translations [41, 48, 54, 55] showed a better fit for a two-factor solution than for a single factor; two found both solutions to fit well [40, 53]; one favored a one-factor solution [44]; and three assessed only the one-factor model [46, 50–52].

### The present study

Up until now, to our knowledge only convenience and selected samples have been used for the Freiburg Mindfulness Inventory (FMI), with no psychometric validation in a representative calibration sample. This raises uncertainty about whether the reported mean values and standard deviations are truly representative for a normal population.

To overcome this gap, given the widespread use of the instrument, we have performed an online study to obtain norm values in a representative German sample. This will allow researchers and clinicians to contextualize individual mindfulness scores. A second objective of our study was to revalidate the scale’s construct validity after almost 20 years, including factorial and predictive validity, ensuring its robustness for practical applications. A third objective was to identify variables in the population that are related to the sum score of the FMI in a regression analysis.

## Method

### Participants

The sampling process aimed at constructing a representative sample of the German population. While sample size is not a necessary condition for representativeness, it is a causal factor for the precision of estimates. To achieve highly accurate parameter estimates, we aimed at receiving approximately 1,000 valid participants. This can be considered a sufficiently large sample to arrive at good precision estimates also for different strata within the sample [56, 57]. To approximate representability, the most important sociodemographic items were used: age (years), gender (m/f/d), highest school education (not finished, basic, GCSE, A-level/high school, university degree, PhD level), living situation (living alone/domestic community with others), religious affiliation (Roman Catholic, Protestant, other Christian denomination, Islam, Buddhism, Judaism, Hinduism, other), permanent employment (yes/no), income situation (monthly net income in Euro: social welfare, 1,000–1,900, 2,000–2,900, 3,000–3,900, 4,000–4,900, above).

### Procedures

The FMI was administered to a participant sample recruited via Debaro GmbH, a professional market research company in Munich with access to approximately 30,000 respondents. Participants are invited to thematic surveys and remunerated accordingly. The company applies stringent quality controls (e.g., IP and proprietary plausibility checks) to prevent double entries. Participants register with the company by providing personal data and interests and are then invited to specific surveys, which they may choose to accept or decline. Participants were offered study participation by the panel. They received a remuneration of 0.50 € for the study participation as part of their overall activity in the panel. They were presented with an initial screen with information about the study, data handling and data protection and had to tick an informed consent statement. Next, they filled in the two instruments (FMI-14 and the PHQ-4) and additional questions (see below and in the preregistered study protocol at https://osf.io/dfzqb/ for the full questionnaire, including wording). The complete assessment took approximately 5–10 min.

### Measures

FMI – short from.

The short form of the Freiburg Mindfulness Inventory (FMI) has 14 items. It uses a four level Likert scale as answer format (anchored at 1=“rarely”, 2=”occasionally”, 3=”fairly often”, 4=”almost always”). Respondents were asked to refer to the last 2 weeks to assess the items. This time frame can also be shortened or lengthened, depending on whether a trait, or a state is the goal of assessment. A sum score can be computed from the 14 items. Additionally, two correlated subscales subfactors: *Presence* (six items) and *Acceptance* (eight items) can be computed.

### PHQ-4

The Patient Health Questionnaire 4 (PHQ-4) is a four-item screening instrument for anxiety and depression and thus of psychological functioning [58, 59]. There are two items each targeting depression and anxiety: “How often did you feel affected by the following complaints during the last 2 weeks? (1) Little interest in doing things; (2) feeling down, depressed or hopeless; (3) feeling nervous, anxious or on edge; (4) not being able to stop or control worrying.” The answer options of this scale are “not at all”, “on single days”, “on more than half of the days”, “almost every day”. The PHQ-4 was included to assess the concurrent criterion validity by means of testing associations of mindfulness as suggested by theory [60–62].

We further asked participants whether they have participated in a meditation, yoga, tai chi, pilates, chi gong, or contemplation class or similar event during the past 5 years (yes – no), whether they engage regularly in any such practice in their daily life (daily - several times a week - about once a week - less often- never). If they did so, we asked for the respective practice (Mindfulness/Vipassana, Zen, Transcendental or Vedanta Meditation, Christian Contemplation, Yoga, Tai Chi, Chi Gong, Tantra, Other; multiple answers were possible) and for how long they were practicing (in years). Finally, we asked whether they participate in longer duration meditation, mindfulness, or contemplation retreats that last at least one weekend (several times per year - once a year - every few years - less often - never) and whether they have already studied the concept of mindfulness theoretically through books, lectures or films (yes – no). These questions were then followed by the sociodemographic questions (see above). All items and questions can be accessed via the study protocol. The study protocol was preregistered at OSF and is available there (https://osf.io/dfzqb/).

### Data analyses

In a first step, items were analyzed descriptively. Second, a classical item analysis, as well as a confirmatory factor analysis were calculated. Third, norm values for different subpopulations are provided. The main analytical work of our contribution consists of a closer look to the factor structure.

Some previous analyses argued for one general factor comprising two highly correlated subfactors. However, such a model faces problems in statistical identification, if no further constraints are imposed on the model such as equal loadings of the lower-order factors on the higher-order factor. We expected that a two-factor model without an overarching general factor would fit the data well.

The prediction based on previous studies [63] that *Presence* would precede *Acceptance* was studied using partial correlation and regression analysis. Internal consistency was reported by McDonald’s Omega rather than Cronbach’s alpha since the former overcomes some shortcomings of the latter [64]. Calculations were performed with Statistica (Version 13) and R (Version 4.2.1). The calculations of the confirmatory factor analysis were performed using the R package lavaan [65]. Given that the scale featured answer options with four categories, we used estimators suited to ordinal level, as advocated in the literature [66], i.e. weighted least squares mean and variance adjusted (WLSMV). One advantage of this estimator (compared to Diagonally Weighted Least Squares, DWLS) is that standard errors are more robust. To assess goodness of fit, we employed indices that align with ordered data, i.e., CFI, TLI, RMSEA, SRMR. In brief, CFI (Comparative Fit Index) and TLI (Tucker-Lewis Index) are relative fit indices comparing the fit of a given model to the null model. The TLI incorporates a penalty for model complexity (in contrast to the CFI). For both indices, higher values (closer to 1) indicate a better fit. Commonly accepted rules of thumb cutoff for good fit is >0.90, with many preferring >0.95 [67]. The RMSEA (Root Mean Square Error of Approximation) and the SRMR (Standardized Root Mean Square Residual) are absolute fit indices. Where the RMSEA investigates the differences between hypothesized model and population covariance matrix, the SRMR draws on the standardized residuals between the covariance of an item *i* and the model-implied covariance of *i*. For both indices, cutoff values range from 0 to 1, with 0 indicating perfect fit. A stringent cutoff for good fit for both RMSEA and SRMR is < 0.05. It is recommended to consider a combination of indices when evaluating model fit [67]. 

To better understand the effect of mindfulness practice on the factorial structure, we also conducted a measurement invariance analysis [68]. A scale shows measurement invariance across subgroups if subjects with the same latent scores have the same raw scores on the measure [69]. Hence, having measurement invariance established is a necessary condition that differences in raw scores between groups can be attributed to the underlying construct. Within structural equation modeling (SEM) a hierarchy of different levels of measurement invariance may be defined: configural, weak, strong, and strict invariance [70, 71]. Each level adds constrains to the previous model. In short, the configural model tests whether the same factor structure holds across groups without imposing equality constraints. The “weak” model assumes that the magnitude of the loadings is similar across the groups. The “strong” model implies that in addition to the loadings the item intercepts are similar across the groups. The “strict” model demands that in addition to the strong model, the residual variances are similar across groups.

The script for the analysis in R (including details on software version and detailed R output) can be accessed at https://osf.io/dfzqb.

## Results

### Descriptive statistics

After prescreening, the final dataset comprised 1.012 participants without missing data. Descriptive data are presented in Table 1 (continuous variables) and Table 2 (frequencies).

The key demographic variables of our sample were similar to those of the German population as a whole. According to the Federal Statistical Office of Germany (https://www.destatis.de, accessed Sept 2nd 2025), 49.35% of the population are male and 50.65% are female. The average age of the population over 18 is 51.5 years, compared to 48.4 years in our sample. In terms of education, our sample had slightly higher levels of education than the general population (basic education 24.4%, GCSE 29.5%, university degree 19.4%, PhD 1.6%).

**Table 1:** Description of the sample: means, minima, maxima, standard deviations (SDs)

Variable	*n*	Mean	SD	Minimum	Maximum
Age	1012	48.40	17.12	15	82
Size of household	1012	2.26	1.22	1	10
Years of Practice	502	6.05	8.40	0	75
PHQ_Sum	1012	7.03	2.89	4	16
PHQ Depression	1012	3.55	1.52	2	8
PHQ Anxiety	1012	3.48	1.60	2	8

**Table 2:** Description of the sample (continued)

Variable	*n*	Percent
Sex:		
Male	495	48.9%
Female	515	50.9%
Diverse	2	0.2%
Education		
Not finished	28	2.8%
Basic	202	20.0%
GCSE	329	32.5%
A-level/high school	196	19.4%
University degree	236	23.3%
PhD level	21	2.1%
Religious affiliation		
Lutheran	260	25.7%
Roman-catholic	231	22.8%
Other Christian	24	2.4%
Islam	55	5.4%
Buddhism	8	0.8%
Hinduism	7	0.7%
Jewish	5	0.5%
Other religion	8	0.8%
None	379	37.4%
Course participation (Yoga, Zen, contemplation, meditation, mindfulness, etc.) during the last 5 years: yes	311	30.7%
Regular practice		
Never	510	50.4%
Rarely	53	5.2%
Once a week	103	10.2%
Several times a week	140	13.8%
Daily	206	20.3%
Regular practice of…		
Mindfulness	220	21.7%
Christian Contemplation	52	5.1%
Zen	48	4.7%
Transcendental Meditation	47	4.6%
Vipassana	33	3.3%
Yoga	212	20.8%
Tai Chi	43	4.2%
Chi Gong	40	3.9%
Tantra	13	1.3%
Other practice*	37	3.6%
Participated in longer term retreats		
Never	673	66.5%
Rarely	79	7.8%
In some years	86	8.5%
Once per year	58	5.7%
Several times per year	116	11.5%
Theoretical study of mindfulness (books, lectures, films)	449	44.4%

*The meaning of “other practice” is mentioned in the supplementary Table S1

Next, we conducted a classical reliability analysis using the R package psych [72] on the full sample (*n* = 1012). The full 14 items version had an omega = 0.87 and a mean item-intercorrelation of r_it_ = 0.32. Item statistics of the FMI-14 are presented in Table S2 in the supplement. Item 13, which is negatively worded and requires recoding due to its negative semantic orientation, stands out as an outlier. It shows low item-scale intercorrelation, and reliability improves when it is excluded. This issue has been noted in previous studies [36, 73]. Therefore, we propose a revised 13-item scale, FMI-13R, which omits the original item 13. The item statistics for this new scale, based on the full sample, are presented in Table 3.

**Table 3:** Item statistics of the FMI-13R scale with item 13 excluded

Nr.	mean	SD	*r*_it_	*r*_it_ dropped
1	1.91	0.85	0.53	0.50
2	1.51	0.89	0.52	0.49
3	1.42	0.86	0.50	0.47
4	1.89	0.86	0.61	0.57
5	1.84	0.83	0.67	0.63
6	1.80	0.82	0.65	0.61
7	1.78	0.84	0.68	0.64
8	1.84	0.77	0.53	0.50
9	1.71	0.84	0.64	0.60
10	1.72	0.85	0.67	0.63
11	1.74	0.84	0.67	0.63
12	1.54	0.86	0.66	0.62
14	1.60	0.84	0.56	0.52

*SD *Standard deviation, *r*_*it*_ Correlation of item with full scale,* r*_*it*_*dropped* Correlation of item with full scale without this item

**Fig. 1:**
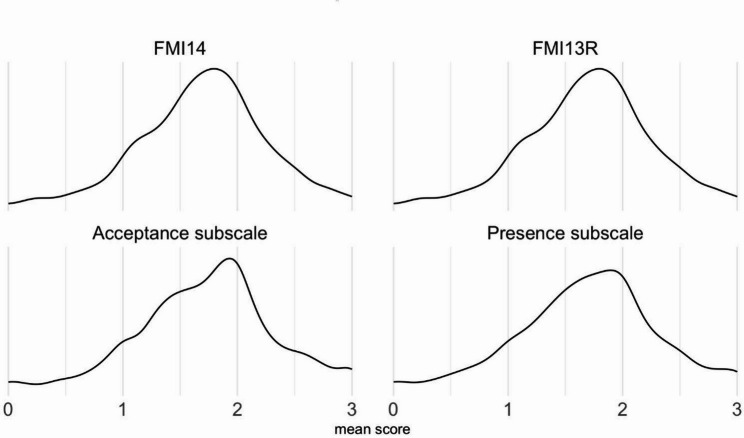
Distribution of the mean values of the FMI-14, FMI-13R, and the Presence and the Acceptance subscales of the FMI-13R. (A more detailed graphical representation is contained in the supplemental material figures S1 - S3, figure S4 presents a normal-probability plot documenting the sufficiently normal distribution.)

FMI-13R exhibits an omega = 0.88 and a mean item-intercorrelation of *r*_*it*_ = 0.36, and thus is psychometrically clearly better than the 14-item version with the negatively worded item 13 included (Table S2). Thus, we propose to use the FMI-13R in the future. We present norms of the mean scores of the full scales in Tables 4 and 5. These were calculated using the full sample (*N* = 1012). Figure 1 depicts the distribution of the mean values of the FMI-14, FMI-13R, together with the distributions of the Presence and the Acceptance subscales.

### Comparison of the factorial structure of FMI-14 and FMI-13R

Next, we validated the factorial structure of the FMI. To that end, we computed a confirmatory factor analysis, comparing the FMI-14 and the FMI-13R. As a robustness analysis, we present the goodness of fit indices for some computational variants for both models. In more detail, we first computed 1) the Maximum Likelihood (ML) estimator (which is the default and applicable to continuous data) as a standard baseline for comparison. Note that ML estimator assumes Pearson type correlation. We compared the ML estimator with the WLSMV estimator which is adequate for ordered (ordinal) data as in the present case. This method builds on polychoric correlations.

**Fig. 2:**
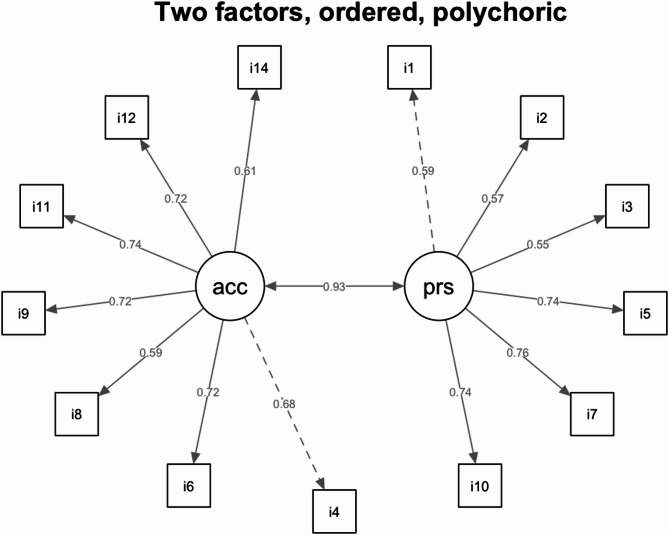
Representation of factor loadings and intercorrelations of factors according to Confirmatory Factor Analysis (model no 6) with the WLSMV estimator based on a polychoric covariance matrix. Standardized coefficients are presented. acc: Acceptance factor; prs: Presence factor

In sum, the model variants assuming ordered data showed more favorable goodness of fit values, see Table 4. In addition, we found that the goodness of fit indices clearly favored the FMI-13R over the FMI-14. Of interest, the goodness of fit was equal for the subsample of mindfulness practitioners and the subsample of nonpractitioners for the FMI-13R (assuming ordinal item level). For the FMI14, the model fit was superior for the subsamples of the practitioners. Converging results were reported for the FFMQ which was replicably shown to have a two-factor higher-order structure [39, 74]. Taken together, results prefer the FMI-13R over the FMI-14. Figure 2 shows model no 6, i.e., FMI-13R based on the ordinal data assumption, using the WLSMV estimator and a polychoric covariance matrix. The correlation between the two subfactors was found to be *r* = 0.93.

**Table 4:** Comparison of model fit statistics for various confirmatory factor analyses for different models. All models assume congeneric measurement (free loadings), if not stated otherwise

Nr	Model	MLR χ²	df	*p*-value	CFI	TLI	RMSEA	SRMR
1	FMI-14, numeric	404.37	77	0.00	0.92	0.91	0.06	0.04
2	FMI-14, ordered	357.41	77	0.00	0.99	0.99	0.06	0.05
3	FMI-14, ordered, practitioners	112.63	77	0.01	1.00	1.00	0.03	0.04
4	FMI-14, ordered, nonpractitioners	310.26	77	0.00	0.98	0.98	0.08	0.07
5	FMI-13R, numeric	280.01	64	0.00	0.95	0.94	0.06	0.04
6	FMI-13R, ordered	228.36	64	0.00	0.99	0.99	0.05	0.04
7	FMI-13R, numeric, practitioners	116.77	64	0.00	0.97	0.97	0.04	0.03
8	FMI-13R, ordered, practitioners	80.98	64	0.07	1.00	1.00	0.02	0.04
9	FMI-13R, numeric, nonpractitioners	251.75	64	0.00	0.92	0.90	0.08	0.05
10	FMI-13R, ordered, nonpractitioners	80.98	64	0.07	1.00	1.00	0.02	0.04
11	FMI-13R, ordered, equal loadings	700.03	75	0.00	0.97	0.97	0.09	0.07
12	FMI-13R, Presence, ordered, congeneric	46.13	9	0.00	0.99	0.99	0.06	0.04
13	FMI-13R, Acceptance, ordered, congeneric	34.58	14	0.00	1.00	1.00	0.04	0.03
14	FMI-13R, Presence, ordered, tau equivalent	99.75	14	0.00	0.98	0.98	0.08	0.05
15	FMI-13R, Acceptance, ordered, tau equivalent	167.53	20	0.00	0.98	0.98	0.09	0.06
16	FMI-13R, Presence, numeric, congeneric	58.23	9	0.00	0.96	0.94	0.07	0.03
17	FMI-13R, Acceptance, numeric, congeneric	49.82	14	0.00	0.98	0.97	0.05	0.02
18	FMI-13R, Presence, numeric, tau equivalent	76.24	14	0.00	0.96	0.95	0.07	0.05
19	FMI-13R, Acceptance, numeric, tau equivalent	113.03	20	0.00	0.95	0.95	0.07	0.08

### Measurement invariance analysis

As the confirmatory factor analysis detected differences between the goodness of fit in the whole sample compared to the subsample of meditation practitioners, we conducted a measurement invariance analysis [68]. Due to the statistical underpinnings of the WLSMV estimator, the parameterization of the model needs to be changed from delta to theta. Likelihood ratio tests were computed along with fit indices to decide about measurement invariance. Results are shown in Table 5.

**Table 5:** Testing the measurement invariance in FMI-13R hierarchy levels. Model 1 was compared against 2, 2 against 3, and 3 against 4. Note: numbers are rounded to two digits

Model	MLR χ²	df	ΔMLR χ²	Δdf	*p*-value	CFI	TLI	RMSEA	SRMR	ΔCFI	ΔRMSEA
1. Configural	298.43	128	-	-	-	0.99	0.99	0.05	0.05	-	-
2. Weak	399.34	139	47.83	11	< 0.001	0.99	0.99	0.06	0.05	0	0.01
3. Strong	470.74	163	90.64	24	< 0.001	0.99	0.99	0.06	0.05	0	0
4. Strict	575.10	176	73.43	13	< 0.001	0.98	0.99	0.07	0.05	−0.01	0.01

As can be seen in Table 5, the Likelihood ratio tests yielded *p*-values below commonly used significance levels, indicating that measurement invariance is to be rejected. However, the loss in goodness of fit is below the commonly used thresholds [75]. In sum, violations to measurement invariance appear negligible, even for strict measurement invariance.

Thus, the factorial structure differs somewhat between meditators and non-meditators. This finding corroborates similar results for the FFMQ [76, 77]. However, the loss in goodness of fit is small as the fit indices revealed.

### Norm values of the FMI-13R

Descriptive statistics of the FMI-13R are shown in Table 6; missing data did not occur. We suggest using these scores for comparison purposes. Table S6-S8 report various norm values (z, Stanine, T, percentage) for the FMI-13R full scale, as well as the two factors (Presence, Acceptance). The full R-output of all these statistical calculations can be accessed at https://osf.io/dfzqb.

**Table 6:** Scale and items distributions for the newly proposed FMI-13R revised scale; mean, standard deviation (*SD*), interquartile range (IQR), minimum, maximum, skewness, kurtosis; in the whole sample and according to group characteristics

	Mean	SD	Minimum	Maximum	Skewness	Kurtosis	Q1	Q3
Total (*n* = 1012)								
FMI-13R	1.71	0.51	0.21	3	−0.15	0.16	1.36	2
Presence	1.70	0.59	0.00	3	−0.24	0.19	1.33	2
Acceptance13	1.73	0.58	0.00	3	−0.22	0.27	1.43	2
Male (*n* = 495)*								
FMI-13R	1.71	0.51	0.21	3	−0.19	0.58	1.43	2
Presence	1.67	0.60	0.00	3	−0.35	0.42	1.33	2
Acceptance13	1.74	0.59	0.00	3	−0.34	0.65	1.43	2
Female (*n* = 515)*								
FMI-13R	1.71	0.51	0.21	3	−0.11	−0.27	1.36	2.07
Presence	1.72	0.58	0.00	3	−0.11	−0.10	1.33	2.0
Acceptance13	1.73	0.57	0.00	3	−0.09	−0.13	1.29	2.07
Regular mindfulness practice (*n* = 220)								
FMI-13R	1.81	0.49	0.21	3	−0.02	0.52	1.50	2.07
Presence	1.86	0.56	0.00	3	−0.20	0.45	1.50	2.17
Acceptance13	1.80	0.55	0.00	3	−0.19	0.64	1.43	2.14
Several retreats per year (*n* = 116)								
FMI-13R	1.82	0.43	0.71	3	0.03	0.32	1.57	2
Presence	1.84	0.50	0.33	3	−0.15	0.28	1.50	2.04
Acceptance13	1.81	0.48	0.71	2	−0.03	−0.06	1.57	2.04
Younger than 49 years (median)								
FMI-13R	1.68	0.59	0.43	2.79	−0.21	0.02	1.37	2.14
Presence	1.68	0.77	0.00	3	−0.37	−0.18	1.33	2.17
Acceptance13	1.70	0.64	0.29	3	−0.02	0.04	1.43	2.14
Older than 49 years (median)								
FMI-13R	1.74	0.51	0.21	3	−0.14	0.16	1.36	2
Presence	1.71	0.59	0.00	3	−0.22	0.19	1.33	2
Acceptance13	1.76	0.58	0.00	3	−0.22	0.28	1.43	2

*SD* Standard deviation, *Q1* Lowest quartile, *Q3* Highest quartile

* n  = 2 diverse gender respondents omitted

The mapping from Stanine values to mean value bands is shown in the Appendix (S9-S11). We observed a paradoxical finding seen before [22], that participants with more experience (participating in several retreats, with regular mindfulness- or Vipassana practice) did not always report higher mindfulness scores (see Table 6 and Supplement Table S5). We presume that this is either due to response shift [78], or to a different understanding of the items between participants with or without regular practice [79]. 

### Support of external validity

The sum of the PHQ-4, which measures anxiety and depression with four items, correlates negatively with the FFA-13R sum score (*r* = −0.15; *p* < 0.0001); this correlation is significant for both the depression subscore of the PHQ-4 (i.e. the first two items summed together) with *r* = − 0.14 (*p* < 0.0001) and for the anxiety subscore (i.e. the second two items of the PHQ-4 summed together) with *r* = − 0.14 (*p* < 0.0001). The overall correlation is driven mainly by Acceptance (*r* = −0.21; *p* < 0.0001), but not by Presence (*r* = −0.06; *p* = 0.06). If Presence is controlled in a partial correlation, the negative correlation between PHQ-4 and Acceptance increases to *r* = −0.25 (*p* < 0.0001). Reversely, if Acceptance is controlled, the correlation between presence and PHQ turns positive into *r* = 0.14 (*p* < 0.001). This finding suggests that the positive impact of mindfulness on anxiety and depression is primarily driven by the Acceptance component, while the Presence component alone may actually be correlated with increased anxiety and depression. Additionally, these findings further support the notion that mindfulness can be conceptually divided into two closely related factors: Presence and Acceptance. This can also be seen in a linear regression analysis of Acceptance and Presence on PHQ-4. Such a model is highly significant (F(2/1.009) = 34.51; *p* = < 0.001) and includes first Acceptance (beta = −0.25) and then Presence (beta = 0.16). It explains 6% of the variance. This result supports previously published findings: Mindfulness has been shown to be a buffering factor against anxiety and depression with mainly the Acceptance facet driving the health relevant effects [63].

The question of whether Acceptance develops as a result of practicing Presence would be best explored in a longitudinal study. However, with our current data, we can only indirectly assess this by comparing individuals with varying meditation practices using regression models. Our findings indicate that the number of years of practice is not a significant predictor for the Patient Health Questionnaire (PHQ-4) scores, suggesting that other factors might play a more crucial role in influencing these outcomes. In contrast, daily practice shows a strong effect on PHQ-4 sum score (beta = −0.31; *p* = 0.01) and an effect of Acceptance (beta = −0.13; *p* < 0.001). The model is highly significant (*p* < 0.001), and explains 3.5% of the variance. To reiterate, our data does not allow for strong conclusions on a causal model as we build on cross-sectional, observational data. However, the present results are promising insofar as they are in line with previous evidence and also in line with the most relevant theoretical model by [36].

### Which variables explain the mindfulness sum score?

In order to clarify which variables are related to the mindfulness sum score, we calculated first a LASSO-regression with all baseline variables as predictors [80]. This so called “Least Absolute Shrinkage Selection Operator“ (LASSO)-Regression is a powerful exploratory tool. It shrinks iteratively all regression weights that do not contribute to explanation to zero, thereby finding out which variables can contribute to explaining variance without calculating multiple models and thereby preventing capitalization on chance. These variables can then be used for a final model. Such a model is presented in Table 7. The full model with all variables identified by the LASSO regression is presented in the supplemental materials (Table S4 and S5). The model is highly significant (F (10,1001) = 13; *p* < 0.0001) and explains roughly 11% of the variance (*R*^*2*^_*adj*_ = 0.11).

**Table 7:** Linear regression to explain the FFA13 sum-score (intercept calculated, but not presented); *n* = 1012

	β (standard error of β)	t-score	*p*
Age	0.15 (0.03)	4.7	< 0.001
Education	0.14 (0.03)	4.5	< 0.001
Religion: Judaism	−0.07 (0.03)	−2.5	0.01
Daily practice	−0.11 (0.04)	−2.7	0.006
Regular mindfulness practice	0.10 (0.03)	2.8	0.004
Yoga regularly	0.18 (0.03)	5.5	< 0.001
Tai Chi regularly	0.07 (0.03)	2.3	0.02
Chi Gong regularly	0.07 (0.03)	2.2	0.03
Years of practice	0.07 (0.03)	2.0	0.048
Theory	0.07 (0.03)	2.0	0.043

The mindfulness sum score is higher in better-educated and older individuals. It is a paradoxical finding that persons with a daily practice have a lower sum score. This might be due to the well-known paradox that experienced practitioners evaluate themselves more critically. Also, followers of Judaism have lower sum scores. This might be due to the fact that Judaism does not encourage meditation, or a consequence on the strictly verbal and language based foundation of this religion [81]. Regular practice of mindfulness, Yoga, Chi Gong, Tai Chi and a longer practice experience are all positively associated with a higher mindfulness sum score, as is theoretical knowledge. In sum, these findings can be seen as supporting the validity of the questionnaire. However, it should be clear that our interpretation can only be suggestive at this point of time given that no experimental and/or longitudinal data is available in the present study.

## Discussion

In this paper, we have presented population-standardized data for the German population for a revised 13-item version (FMI-13R), removing the negatively worded item 13. The shortened instrument is psychometrically sound, with one general factor, which can be conceptually subdivided into two subfacets, Presence and Acceptance. Acceptance correlates negatively with depression and anxiety, while Presence correlates positively when controlled for Acceptance. A Confirmatory Factor Analysis supports this distinction into two facets with a reasonably good fit. A regression analysis supports the validity of the instrument: the mindfulness score is higher in those participants who practice more and who are older. Interestingly, experienced practitioners sometimes report lower scores, probably due to a response shift, where deeper insights lead them to become more critical of their self-assessed mindfulness levels. Response shift is a bias where those who are very diligent in their practice become more critical of their state of mindfulness [82].

We have confirmed that the original item 13, which is the only negatively worded item (and therefore has to be recoded; “I am impatient with myself and others”) is, psychometrically spoken, an outlier. This item was excluded because, as a negatively worded item, it consistently showed poor loadings and impaired model fit. While such wording effects were not yet fully recognized when the FMI was developed in the early 2000 s, subsequent later psychometric research has demonstrated that reverse-coded items often introduce method variance and reduce reliability [83, 84]. Removing item 13, thus improves both the consistency and structural validity of the scale.

The model performance values in the present study are generally somewhat lower than in other studies such as those by [34, 36, 85–87]. This has very likely to do with the fact that we have an unselected sample representative of the German population, while most mindfulness studies attract persons with some affinity to the concept of mindfulness and/or spirituality. This increases variance and reduces the goodness-of-fit of models. Hence, the results of this study may provide a more robust and more realistic picture compared to many other studies.

Our analysis confirms that mindfulness is a global construct. One may, however, for purposes of clarification or for conceptual issues, separate it into highly correlated subconstructs, Presence, and Acceptance. Over and above, previous research reached similar conclusions as to the factorial structure [36, 87]. This result regarding the factor structure of the FMI has also some pragmatic consequences. Our findings suggest that the Acceptance facet is more important for clinical and health effects, such as reduced anxiety or depression, but that in order to grow acceptance, a practice of presence is useful [63]. In a similar vein, a recent study reports that Acceptance, and not Presence, increases psychological well-being [4], thus corroborating our finding. As this is the result of interpreting our cross-sectional data, this needs to be supported by longitudinal studies that can map the temporal development of practitioners. It should be noted that other self-assessment scales of mindfulness tend to conceptualize subfactors similar to Presence and Acceptance, too [88–91]. An exception is the Langer Mindfulness Scale [88, 92–94], which contextualizes mindfulness predominantly in the context of Western psychology, where mindfulness is defined as an attitude of openness to novelty in which the individual draws novel distinctions, i.e., constructs her or his own categories.

The results of a measurement invariance analysis, albeit mixed, provide confidence that strong or even strict invariance holds in the FMI13R, over and above. If measurement invariance is shown, then the scale is expected to work similarly between specific populations, such as mindfulness practitioners and non-practitioners.

Our factorial analysis replicates the factorial structure as put forward by [36]. We have also analyzed this previous allocation of items to constructs and obtained a satisfactory fit with these data. Depending on the sample, a model fit of a confirmatory model supports either allocation of items to constructs. We have chosen here to report the previous allocation of items to constructs, because the model fit statistics allow this even for the general sample. So, depending on which kind of individuals or sample are measured, with or without meditation practice, practitioners may feel free to use either model, if they want to investigate subdomains of the global mindfulness construct.

While the factorial structure remains, over and above, the same compared to previous studies, the question why there are some differences in the factor structure found, can be answered by the fact that we have used a general, representative sample. If this is used, a slightly different item-allocation ensues. The previously found structure is useful for a sample or individuals with meditative, spiritual or contemplative practice.

Compared to the vast majority of similar studies, we built on a general-population representative study. In contrast, many other studies tap into convenience samples or samples of mindfulness practitioners. In this light, it seems plausible that results might differ somewhat thereby reflecting the (slightly) diverging mental constructs of the participants. However, it seems reassuring to us that the factorial structures are similar to a large extent. As the present analysis is derived from a general population sample, it is likely more representative and robust.

While it’s true that several well-supported instruments for measuring self-attributed mindfulness levels exist, such as the widely used Five Facets Mindfulness Questionnaire, this study addresses a key gap. Most existing research relies on convenience samples like patients, students, or practitioners, rather than representative samples. The FMI-13R offers a concise yet reliable measure of mindfulness, widely used across different languages and cultures for nearly 20 years. We believe that the field would benefit from more studies using representative samples, providing a more accurate gauge of individual mindfulness levels in different cultural contexts. One might argue against the usage of any questionnaire instruments to assess mindfulness [22]. For example, a recent study advocates breath counting as a measure of mindfulness [23]. One further behavioral alternative to self-assessment, perceptual stability in perceiving the Necker Cube, has been elaborated on earlier [95]. While we agree that self-reported mindfulness levels may not be the optimal way to assess mindfulness, especially given potentially occurring response shift phenomena also observed in this study, it remains a pragmatic approach. This method is well-supported by empirical evidence, as highlighted in several reviews [20, 21, 88]. Ideally, one would have various and multi-level-analyses methods, such as observation of actions and daily practice in addition to standardized self-report instruments. This is the traditional testing mode in, for instance spiritual traditions using breath awareness such as Zen and Vipassana practice [23, 96]. However, such mindfulness assessments might only be valuable in contexts focusing on personality development or spiritual growth. For such purposes, we do not currently recommend relying solely on any questionnaire instrument. But if, for instance, a clinician wants to get an overview over various personality components in a client or patient, or if researchers want to assess progress of participants in a mindfulness training program, questionnaires are still a parsimonious and robust method of collecting data. It was in this spirit that we conducted this study and present population norm data representative for the present population in Germany almost twenty years after the inception of the FMI.

The primary objective of this study is to provide norm values and robust statistics derived from a representative sample, but our findings also offer insights into potential pathways through which mindfulness exerts its effects. Notably, while much research focuses on the effects of mindfulness and its measurement, less attention has been given to how mindfulness works (but see [97–101]). Our results suggest that Acceptance, rather than Presence, mediates the beneficial effects of mindfulness. We hypothesize that accepting negatively valenced experiences may directly alleviate health and distress conditions, while presence serves to build acceptance. This might also explain, why anecdotal evidence also speaks of initial aggravations of anxiety or depression: when mindfulness is first practiced, the presence of mind might highlight the actual severity of a problematic situation, and only if and when acceptance is built up will this change. This is clearly a hypothesis that warrants further scrutiny, but might help leaders of mindfulness groups and practitioners to overcome obstacles, by shifting emphasis gradually to acceptance.

This insight could also inform future mindfulness interventions and potentially explore other methods, such as neurofeedback, to enhance individual acceptance levels. The strength of this study is the fact that we used an approximation to the German population by employing a professional response panel and approximating the most important sociodemographic parameters of the German population. Thus, there is no positive selection bias due to affinity to meditation, mindfulness or other spiritual practices. This also resulted in a comparatively large sample of more than 1.000 participants. We thus are able to report population standardized scores which should allow researchers and practitioners to use the instrument also for assessment purposes, where mindfulness is to be assessed, for instance for purposes of comparison, or for the assessment of development.

### Limitations and future research

A word of caution is due: Every self-assessment instrument is prone to a social desirability bias [102, 103]. Indirect hints that this might be a problem can be seen in our regression analysis, where a response shift becomes visible: whoever has theoretical knowledge of mindfulness has in tendency a higher score. Conversely, practitioners with more practice and experience tend to score lower, likely because they assess themselves more critically. This effect is well-known and has been dubbed the Dunning-Kruger Effect [104]. These are some of the limitations of such instruments in general. If these limitations are kept in mind, one would use scores derived from such instruments diligently, i.e. not in an absolute and definitive way, but as an indicator which might be most useful if assessed in comparison to other indicators or in its temporal sequence.

Another reason for our paradoxical findings might be semantic issues: We have long observed that meditation practitioners and naïve individuals who have never heard of mindfulness understand various items differently [79]. Thus, practitioners might have a much more varied and also critical understanding of, say, “I am accepting myself when things go wrong”, than do naïve individuals. Such semantic opacity is part and parcel of the questionnaire methodology, no matter what construct is to be assessed. Mindfulness is no exception. Hence our previous caution to use the instrument wisely within certain delimited contexts, and if absolutely robust assessments of mindfulness are to be made to use some multilevel approach, where behavioral or physiological data are included as well.

Although we tried to achieve representativity using a professional panel, our sample is not fully representative. To achieve this, a complex sampling routine needs to be followed using interviews and stratified samples covering cities, countryside regions and respecting other aspects. Such a strategy is very costly and would have exceeded the budget of this self-funded study. However, using a panel as we did we could at least approach representativity, as the comparison with important markers of the average German population according to official Federal statistics, which we have reported above.

It might be useful to explore in future studies, how the two components, Presence and Acceptance, are related in their temporal development and how they relate to other relevant constructs. Moreover, as is standard in clinical assessment, the mindfulness field would benefit from more studies using representative or diverse samples, like the present study.

A clearer understanding of mindfulness’s causal mechanisms and how they develop will, in turn, enhance measurement methods. The better we understand what mindfulness does to the body and mind, the more effectively we can measure these effects. Specifically, Presence and Acceptance might be particularly suited to behavioral and physiological measurements. For example, a recent study investigated experience-sampling in the context of breathing concentration exercise as behavioral means for measuring mindfulness [23]. Relatedly, perceptual stability of ambiguous images may prove to serve as a behavioral measurement of mindfulness [95]. Furthermore, our results suggest that Acceptance as a mediator of change is a worthwhile candidate of research efforts, in line with existing evidence [62, 101, 105]. We suggest that future studies continue to elaborate this direction.

In conclusion, the newly revised 13-item version of the Freiburg Mindfulness Inventory (FMI-13R) is a psychometrically robust tool for assessing self-attributed mindfulness, validated in a representative German population sample. It can be applied for both short-term state and long-term trait assessments, depending on the specified time frame. While it measures a general mindfulness factor, this can be further divided into two correlated components: Presence and Acceptance, supported by CFA and partial correlations. The population-standardized data provided here helps to contextualize findings using this instrument.

## Supplementary Information

Supplementary Material 1.

## Data Availability

All data are available at https://osf.io/dfzqb/.

## Abbreviations

CFA: Confirmatory Factor Analysis
CFI: Comparative Fit Index
DWLS: Diagonally Weighted Least Squares
FFA: Freiburger Fragebogen zur Achtsamkeit
FFMQ: Five Facts Mindfulness Questionnaire
FMI: Freiburg Mindfulness Inventory
FMI-13R: Freiburg Mindfulness Inventory revised 13-item version
GCSE: General Certificate of Secondary Education
IQR: Interquartile Range
LASSO: Least Absolute Shrinkage Selection Operator
ML: Maximum Likelihood
PHQ-4: Patient Health Questionnaire
RMSEA: Root Mean Square Error of Approximation
SD: Standard Deviation
SEM: Structural Equation Modeling
SRMR: Standardized Root Mean Square Residual
TLI: Tucker-Lewis Index
WLSMV: Weighted Least Squares Mean and Variance adjusted
