# Supplementary material for: Freiburg mindfulness inventory (FMI) short form and revised form (FMI-13R) — norm scores and psychometrics in a representative German sample
Source: BMC Psychol. 2025 Nov 26;13:1328. doi: 10.1186/s40359-025-03671-3 (PMC12670850; doi:10.1186/s40359-025-03671-3)
Supplement: Supplementary file 1 — Supplementary Material 1. [file 40359_2025_3671_MOESM1_ESM.docx]

Supplement

**Supplementary Figures and Tables**

**
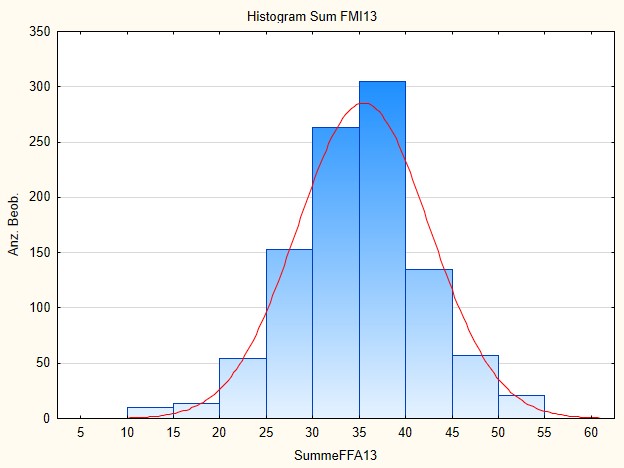
**

Figure S1 – Histogram of the Sum of FMI-13R


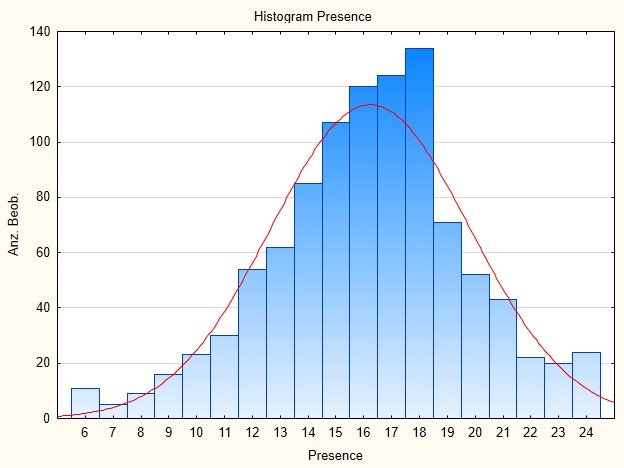


Figure S2 – Histogram of Subscale Presence of FMI_13R


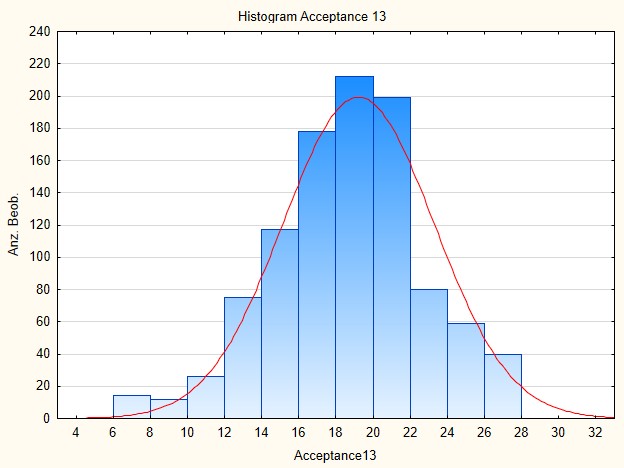


Figure S3 – Histogram of Subscale Acceptance of FMI-13R


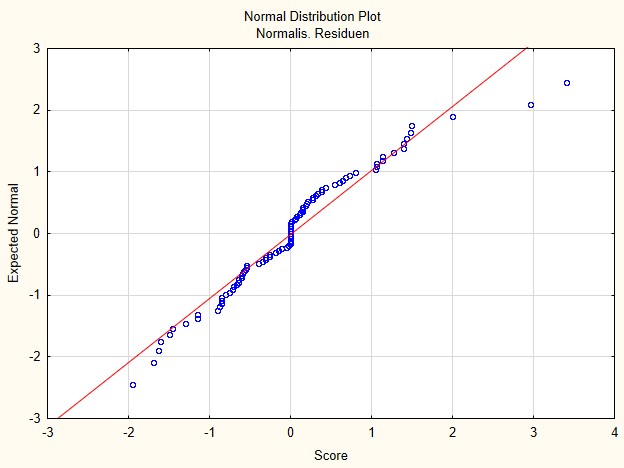


Figure S4 – Normalprobability Plot of the residuals of the confirmatory factor analysis model

Table S1 – Single indications of „regular practice“

| Indication | n | Prozent |
| --- | --- | --- |
| none | 1 | 0,1% |
| contemplating | 1 | 0,1% |
| meditation | 1 | 0,1% |
| Cranio Sacral | 1 | 0,1% |
| Archery | 1 | 0,1% |
| Biking or hiking in nature | 1 | 0,1% |
| Guided meditation | 1 | 0,1% |
| Body Scan | 1 | 0,1% |
| Stress breathing techniques | 1 | 0,1% |
| Nothing | 1 | 0,1% |
| Concentration exercises | 1 | 0,1% |
| Dancing | 1 | 0,1% |
| Autogenic Training, Relaxation | 1 | 0,1% |
| Boxing | 2 | 0,2% |
| Quiet thinking about various topics | 3 | 0,3% |
| Own relaxation exercises | 1 | 0,1% |
| fitness training | 1 | 0,1% |
| Relaxation techniques | 1 | 0,1% |
| Progressive muscle relaxation according to Jacobson | 1 | 0,1% |
| Relaxation | 1 | 0,1% |
| Nothing | 1 | 0,1% |
| Quiet music | 1 | 0,1% |
| Consuming 5-MeO-Mipt | 1 | 0,1% |
| Relaxation according to Jacobson | 1 | 0,1% |
| Autogenic Training | 1 | 0,1% |
| Pilates | 1 | 0,1% |
| Autogenicttraining | 1 | 0,1% |
| 5 prayers per day | 1 | 0,1% |
| Conversation in texts | 1 | 0,1% |

Table S2 – Item statistics of the full FMI14 scale with negatively worded item 13 included

| **ID** | **Label** | **Facet** | **Mean** | **SD** | **Skewness** | **Kurtosis** |
| --- | --- | --- | --- | --- | --- | --- |
| 1 | I am open to the experience of the present moment. | Presence | 1.91 | 0.85 | -0.52 | -0.24 |
| 2 | I sense my body, whether eating, cooking, cleaning or talking. | Presence | 1.51 | 0.89 | -0.01 | -0.73 |
| 3 | When I notice an absence of mind, I gently return to the experience of the here and now. | Presence | 1.42 | 0.86 | 0.03 | -0.66 |
| 4 | I am able to appreciate myself. | Acceptance | 1.89 | 0.86 | -0.4 | -0.48 |
| 5 | I pay attention to what’s behind my actions. | Presence | 1.84 | 0.83 | -0.43 | -0.29 |
| 6 | I see my mistakes and difficulties without judging them. | Acceptance | 1.8 | 0.82 | -0.28 | -0.43 |
| 7 | I feel connected to my experience in the here-and-now. | Presence | 1.78 | 0.84 | -0.39 | -0.37 |
| 8 | I accept unpleasant experiences. | Acceptance | 1.84 | 0.77 | -0.29 | -0.27 |
| 9 | I am friendly to myself when things go wrong. | Acceptance | 1.71 | 0.84 | -0.27 | -0.46 |
| 10 | I watch my feelings without gelng lost in them. | Presence | 1.72 | 0.85 | -0.3 | -0.49 |
| 11 | In diﬃcult situations, I can pause without immediately reacting. | Acceptance | 1.74 | 0.84 | -0.28 | -0.47 |
| 12 | I experience moments of inner peace and ease, even when things get hectic and stressful. | Acceptance | 1.54 | 0.86 | -0.07 | -0.65 |
| 13 | I am impatient with myself and with others.* | Acceptance | 1.67 | 0.9 | -0.15 | -0.77 |
| 14 | 4 I am able to smile when I notice how I sometimes make life diﬃcult. | Acceptance | 1.6 | 0.84 | -0.14 | -0.54 |

*Notes*. No missing values in all items. Min=0 and Max=3 for each item. * Item 13 is negatively worded, needs recoding; dropped for FMI13-R.*

Table S3 – Mean scores over the sums for the original FMI-14 scale and revised 13 item scale FMI-13R; mean, standard deviation (*SD*), interquartile range (IQR), minimum, maximum, skewness, kurtosis

|  | Mean | *SD* | IQR | Minimum | Maximum | Skewness | Kurtosis |
| --- | --- | --- | --- | --- | --- | --- | --- |
| Total (*n* = 1012) |  |  |  |  |  |  |  |
| Sum FMI-14 | 37.99 | 7.13 | 9 | 17 | 56 | -0.15 | 0.16 |
| Sum FMI-13R | 35.32 | 7.08 | 9 | 13 | 52 | -0.21 | 0.39 |
| Sum Presence* | 16.18 | 3.56 | 4 | 6 | 24 | -0.24 | 0.19 |
| Sum Acceptance | 21.80 | 4.20 | 5 | 9 | 32 | -0.11 | -0.01 |
| Sum Accpetance13** | 19.14 | 4.05 | 4 | 7 | 28 | -0.22 | 0.27 |

*SD* = standard deviation; IQR = interquartile range; *for the shorter version FMI-13R the items contributing to presence are the same as for FMI-14; ** Acceptance scale for FMI-13R, dropping negatively worded original item no. 13.

Table S4 - Lasso Regression: Number of iterations (24), Lambda parameters, variables and their weight after each iteration step

| Sum FMI13 – Matrix of coefficients (Achtsamkeit_Daten_FFAEichung_rekodiert) Linear Regression | | | | | | | | | | | | | | | | |  |  |  |  |  |  |  |  |  |  |  |
| --- | --- | --- | --- | --- | --- | --- | --- | --- | --- | --- | --- | --- | --- | --- | --- | --- | --- | --- | --- | --- | --- | --- | --- | --- | --- | --- | --- |
|  | \| Lambda \| \| --- \| | \| FG \| \| --- \| | \| Kon-stante \| \| --- \| | \| Age \| \| --- \| | \| Edu-cation \| \| --- \| | \| Size of house-hold \| \| --- \| | \| employed \| \| --- \| | \| In-come \| \| --- \| | \| course \| \| --- \| | \| Daily practice \| \| --- \| | \| Mindf. Reg. \| \| --- \| | \| Vip-  regl \| \| --- \| | \| Zen-reg. \| \| --- \| | \| TM-regl \| \| --- \| | \| Contemp regl \| \| --- \| | \| Yogaregl \| \| --- \| | \| Vip_regl \| \| --- \| | \| Zen_reg. \| \| --- \| | \| TM_regl \| \| --- \| | \| Contemp regl \| \| --- \| | \| Yoga_regl \| \| --- \| | \| TaiChi_regl \| \| --- \| | \| ChiGong_regl \| \| --- \| | \| Tantra_regl \| \| --- \| | \| Other_regl \| \| --- \| | \| Years of practice \| \| --- \| | \| Retreats \| \| --- \| |
| \| 1 \| \| --- \| | 1,30 | 0 | 35,32 |  |  |  |  |  |  |  |  |  |  |  |  |  |  |  |  |  |  |  |  |  |  |  |  |
| \| 2 \| \| --- \| | 1,18 | 2 | 34,97 |  | 0,09 |  |  |  |  |  |  |  |  |  |  | 0,21 |  |  |  |  | 0,21 |  |  |  |  |  |  |
| \| 3 \| \| --- \| | 1,08 | 2 | 34,65 |  | 0,17 |  |  |  |  |  |  |  |  |  |  | 0,44 |  |  |  |  | 0,44 |  |  |  |  |  |  |
| \| 4 \| \| --- \| | 0,98 | 2 | 34,36 |  | 0,24 |  |  |  |  |  |  |  |  |  |  | 0,65 |  |  |  |  | 0,65 |  |  |  |  |  |  |
| \| 5 \| \| --- \| | 0,90 | 2 | 34,10 |  | 0,30 |  |  |  |  |  |  |  |  |  |  | 0,84 |  |  |  |  | 0,84 |  |  |  |  |  |  |
| \| 6 \| \| --- \| | 0,82 | 3 | 33,77 | 0,00 | 0,36 |  |  |  |  |  |  |  |  |  |  | 1,02 |  |  |  |  | 1,02 |  |  |  |  |  |  |
| \| 7 \| \| --- \| | 0,74 | 4 | 33,27 | 0,01 | 0,41 |  |  |  |  |  |  |  |  |  |  | 1,20 |  |  |  |  | 1,20 |  |  |  |  |  |  |
| \| 8 \| \| --- \| | 0,68 | 6 | 32,78 | 0,01 | 0,45 |  |  |  |  |  |  |  |  |  |  | 1,35 |  |  |  |  | 1,35 |  | 0,24 |  |  | 0,00 |  |
| \| 9 \| \| --- \| | 0,62 | 8 | 32,33 | 0,02 | 0,48 |  |  | 0,01 |  |  | 0,03 |  |  |  |  | 1,47 |  |  |  |  | 1,47 |  | 0,46 |  |  | 0,01 |  |
| \| 10 \| \| --- \| | 0,56 | 8 | 31,90 | 0,02 | 0,50 |  |  | 0,03 |  |  | 0,12 |  |  |  |  | 1,57 |  |  |  |  | 1,57 |  | 6 |  |  | 0,01 |  |
| \| 11 \| \| --- \| | 0,51 | 9 | 31,51 | 0,03 | 0,52 |  |  | 0,045 |  |  | 0,20 |  |  |  |  | 1,66 |  |  |  |  | 1,66 |  | 4 |  |  | 0,01 |  |
| \| 12 \| \| --- \| | 0,47 | 9 | 31,17 | 0,03 | 0,53 |  |  | 0,06 |  |  | 0,27 |  |  |  |  | 1,74 |  |  |  |  | 1,74 |  | 1,00 |  |  | 0,01 |  |
| \| 13 \| \| --- \| | 0,43 | 11 | 30,85 | 0,03 | 0,55 |  |  | 0,08 |  |  | 0,34 |  |  |  |  | 1,81 |  |  |  |  | 1,81 | 0,10 | 1,13 | 0,06 |  | 0,02 |  |
| \| 14 \| \| --- \| | 0,39 | 12 | 30,54 | 0,03 | 0,56 |  |  | 0,09 |  |  | 0,41 |  |  |  |  | 1,88 |  |  |  |  | 1,88 | 0,26 | 1,24 | 0,36 |  | 0,0 |  |
| \| 15 \| \| --- \| | 0,35 | 12 | 30,26 | 0,037 | 0,57 |  |  | 0,11 |  |  | 0,46 |  |  |  |  | 1,94 |  |  |  |  | 1,94 | 0,41 | 1,34 | 0,64 |  | 0,02 |  |
| \| 16 \| \| --- \| | 0,32 | 12 | 30,00 | 0,04 | 0,58 |  |  | 0,12 |  |  | 0,52 |  |  |  |  | 2,00 |  |  |  |  | 2,00 | 0,55 | 1,43 | 0,89 |  | 0,02 |  |
| \| 17 \| \| --- \| | 0,29 | 12 | 29,77 | 0,04 | 0,58 |  |  | 0,13 |  |  | 0,56 |  |  |  |  | 2,04 |  |  |  |  | 2,04 | 0,67 | 1,52 | 1,12 |  | 0,03 |  |
| \| 18 \| \| --- \| | 0,27 | 14 | 29,56 | 0,04 | 0,59 |  |  | 0,14 |  |  | 0,61 |  |  |  | 0,04 | 2,09 |  |  |  | 0,04 | 2,09 | 0,78 | 1,59 | 1,33 |  | 0,03 |  |
| \| 19 \| \| --- \| | 0,24 | 15 | 29,37 | 0,05 | 0,60 |  |  | 0,15 |  |  | 0,64 |  | -0,06 |  | 0,12 | 2,14 |  | -0,06 |  | 0,12 | 2,14 | 0,89 | 1,64 | 1,54 |  | 0,03 |  |
| \| 20 \| \| --- \| | 0,22 | 16 | 29,21 | 0,05 | 0,61 |  |  | 0,15 |  | 0,00 | 0,68 |  | -0,19 |  | 0,20 | 2,19 |  | -0,19 |  | 0,20 | 2,19 | 0,99 | 1,69 | 1,73 |  | 0,03 |  |
| \| 21 \| \| --- \| | 0,20 | 17 | 29,10 | 0,05 | 0,62 |  |  | 0,16 |  | -0,05 | 0,76 |  | -0,33 |  | 0,29 | 2,28 |  | -0,33 |  | 0,29 | 2,28 | 1,11 | 1,75 | 1,90 |  | 0,03 | 0,01 |
| \| 22 \| \| --- \| | 0,18 | 18 | 29,02 | 0,05 | 0,62 |  |  | 0,16 | -0,06 | -0,1 | 0,85 |  | -0,44 |  | 0,38 | 2,38 |  | -0,44 |  | 0,38 | 2,38 | 1,23 | 1,81 | 2,05 |  | 0,03 | 0,03 |
| \| 23 \| \| --- \| | 0,17 | 19 | 28,92 | 0,05 | 0,63 |  |  | 0,17 | -0,16 | -0,13 | 0,93 |  | -0,53 |  | 0,46 | 2,48 |  | -0,53 |  | 0,46 | 2,48 | 1,35 | 1,89 | 2,18 |  | 0,04 | 0,05 |
| \| 24 \| \| --- \| | 0,15 | 21 | 28,83 | 0,05 | 0,64 |  |  | 0,18 | -0,24 | -0,17 | 1,00 |  | -0,62 |  | 0,54 | 2,57 |  | -0,62 |  | 0,54 | 2,57 | 1,47 | 1,95 | 2,31 |  | 0,04 | 0,07 |

Continued to the right on the next page

|  | SummeFFA13 - Koeffizientenmatrix (Achtsamkeit_Daten_FFAEichung_rekodiert) Lineare Regression | | | | | | | | | | | | |
| --- | --- | --- | --- | --- | --- | --- | --- | --- | --- | --- | --- | --- | --- |
|  | \| Theory \| \| --- \| | \| male \| \| --- \| | \| female \| \| --- \| | \| diverse \| \| --- \| | \| catholic \| \| --- \| | \| protestant \| \| --- \| | \| Other Christian \| \| --- \| | \| Islam \| \| --- \| | \| Buddhism \| \| --- \| | \| Judais \| \| --- \| | \| Hinduism \| \| --- \| | \| Other religion \| \| --- \| | \| No religion \| \| --- \| |
| \| 1 \| \| --- \| |  |  |  |  |  |  |  |  |  |  |  |  |  |
| \| 2 \| \| --- \| |  |  |  |  |  |  |  |  |  |  |  |  |  |
| \| 3 \| \| --- \| |  |  |  |  |  |  |  |  |  |  |  |  |  |
| \| 4 \| \| --- \| |  |  |  |  |  |  |  |  |  |  |  |  |  |
| \| 5 \| \| --- \| |  |  |  |  |  |  |  |  |  |  |  |  |  |
| \| 6 \| \| --- \| |  |  |  |  |  |  |  |  |  |  |  |  |  |
| \| 7 \| \| --- \| | 0,05 |  |  |  |  |  |  |  |  |  |  |  |  |
| \| 8 \| \| --- \| | 0,17 |  |  |  |  |  |  |  |  |  |  |  |  |
| \| 9 \| \| --- \| | 0,25 |  |  |  |  |  |  |  |  |  |  |  |  |
| \| 10 \| \| --- \| | 0,32 |  |  |  |  |  |  |  |  |  |  |  |  |
| \| 11 \| \| --- \| | 0,38 |  |  |  |  |  |  |  |  | -0,38 |  |  |  |
| \| 12 \| \| --- \| | 0,44 |  |  |  |  |  |  |  |  | -0,97 |  |  |  |
| \| 13 \| \| --- \| | 0,48 |  |  |  |  |  |  |  |  | -1,52 |  |  |  |
| \| 14 \| \| --- \| | 0,52 |  |  |  |  | 0,06 |  |  |  | -2,01 |  |  |  |
| \| 15 \| \| --- \| | 0,55 |  |  |  |  | 0,13 |  |  |  | -2,46 |  |  |  |
| \| 16 \| \| --- \| | 0,58 |  |  |  |  | 0,20 |  |  |  | -2,87 |  |  |  |
| \| 17 \| \| --- \| | 0,61 |  |  |  |  | 0,26 |  |  |  | -3,24 |  |  |  |
| \| 18 \| \| --- \| | 0,64 |  |  |  |  | 0,30 |  |  |  | -3,58 |  |  | -0,01 |
| \| 19 \| \| --- \| | 0,66 |  |  |  |  | 0,33 |  |  |  | -3,90 |  |  | -0,05 |
| \| 20 \| \| --- \| | 0,68 |  |  |  |  | 0,35 |  |  |  | -4,15 |  |  | -0,08 |
| \| 21 \| \| --- \| | 0,70 |  |  |  |  | 0,38 |  |  |  | -4,38 |  |  | -0,11 |
| \| 22 \| \| --- \| | 0,73 |  |  |  |  | 0,39 |  |  |  | -4,59 |  |  | -0,14 |
| \| 23 \| \| --- \| | 0,77 |  |  |  |  | 0,42 |  | 0,09 |  | -4,76 |  |  | -0,16 |
| \| 24 \| \| --- \| | 0,81 | -0,04 | 0,00 |  |  | 0,45 |  | 0,21 |  | -4,88 |  |  | -0,17 |

Table S5 – Linear Regression on FFA13 sum score of all variables, that a LASSO regression (Table S5) had identified as potential predictors. R^2^_adj_ = 0.11, F (18.993) = 8,4; p < .001

|  | *Beta (std. error beta)* | *t-score* | *p* |
| --- | --- | --- | --- |
| Age | 0.14 (0.03) | 4.2 | 0.00 |
| Education | 0.12 (0.03) | 3.4 | 0.00 |
| Lutheran religion | 0.04 (0.03) | 1.3 | 0.20 |
| Islam | 0.05 (0.03) | 1.4 | 0.16 |
| Judaism | -0.06 (0.03) | -2.1 | 0.03 |
| No religion | -0.02 (0.04) | -0.6 | 0.55 |
| Income | 0.05 (0.03) | 1.6 | 0.11 |
| Course participation | -0.06 (0.04) | -1.7 | 0.09 |
| Daily practice | -0.11 (0.04) | -2.8 | 0.06 |
| Mindfulness regularly | 0.10 (0.03) | 3.0 | 0.00 |
| Zen regularly | -0.04 (0.03) | -1.4 | 0.16 |
| Contemplation regularly | 0.04 (0.03) | 1.4 | 0.16 |
| Yoga regularly | 0.21 (0.03) | 5.8 | 0.00 |
| TaiChi regularly | 0.08 (0.03) | 2.5 | 0.01 |
| ChiGong regularly | 0.07 (0.03) | 2.2 | 0.02 |
| Tantra regularly | 0.06 (0.03) | 1.9 | 0.05 |
| Years of practice | 0.06 (0.03) | 1.8 | 0.06 |
| Theory | 0.09 (0.03) | 2.4 | 0.01 |

Table S6 – Norm values for the FMI-13R

| **Mean** | **Percent (empirical)** | **z** | **Stanine** | **T** | **Percent (normal)** |
| --- | --- | --- | --- | --- | --- |
| 0.0 | 0.00 | -3.37 | 1.00 | 16.35 | 0.00 |
| 0.1 | 0.00 | -3.17 | 1.00 | 18.31 | 0.00 |
| 0.2 | 0.00 | -2.97 | 1.00 | 20.28 | 0.00 |
| 0.3 | 0.01 | -2.78 | 1.00 | 22.24 | 0.00 |
| 0.4 | 0.01 | -2.58 | 1.00 | 24.21 | 0.00 |
| 0.5 | 0.02 | -2.38 | 1.00 | 26.17 | 0.01 |
| 0.6 | 0.02 | -2.19 | 1.00 | 28.13 | 0.01 |
| 0.7 | 0.03 | -1.99 | 1.02 | 30.10 | 0.02 |
| 0.8 | 0.04 | -1.79 | 1.41 | 32.06 | 0.04 |
| 0.9 | 0.05 | -1.60 | 1.80 | 34.02 | 0.06 |
| 1.0 | 0.09 | -1.40 | 2.20 | 35.99 | 0.08 |
| 1.1 | 0.12 | -1.20 | 2.59 | 37.95 | 0.11 |
| 1.2 | 0.15 | -1.01 | 2.98 | 39.92 | 0.16 |
| 1.3 | 0.21 | -0.81 | 3.38 | 41.88 | 0.21 |
| 1.4 | 0.25 | -0.62 | 3.77 | 43.84 | 0.27 |
| 1.5 | 0.34 | -0.42 | 4.16 | 45.81 | 0.34 |
| 1.6 | 0.40 | -0.22 | 4.55 | 47.77 | 0.41 |
| 1.7 | 0.45 | -0.03 | 4.95 | 49.74 | 0.49 |
| 1.8 | 0.58 | 0.17 | 5.34 | 51.70 | 0.57 |
| 1.9 | 0.64 | 0.37 | 5.73 | 53.66 | 0.64 |
| 2.0 | 0.76 | 0.56 | 6.13 | 55.63 | 0.71 |
| 2.1 | 0.80 | 0.76 | 6.52 | 57.59 | 0.78 |
| 2.2 | 0.83 | 0.96 | 6.91 | 59.56 | 0.83 |
| 2.3 | 0.89 | 1.15 | 7.30 | 61.52 | 0.88 |
| 2.4 | 0.91 | 1.35 | 7.70 | 63.48 | 0.91 |
| 2.5 | 0.95 | 1.54 | 8.09 | 65.45 | 0.94 |
| 2.6 | 0.96 | 1.74 | 8.48 | 67.41 | 0.96 |
| 2.7 | 0.97 | 1.94 | 8.88 | 69.38 | 0.97 |
| 2.8 | 0.99 | 2.13 | 9.00 | 71.34 | 0.98 |
| 2.9 | 0.99 | 2.33 | 9.00 | 73.30 | 0.99 |
| 3.0 | 1.00 | 2.53 | 9.00 | 75.27 | 0.99 |

Table S7 – Norm values for the FMI-13R, *Presence* factor

| **Mean** | **Percent (empirical)** | **z** | **Stanine** | **T** | **Percent (normal)** |
| --- | --- | --- | --- | --- | --- |
| 0.0 | 0.01 | -2.86 | 1.00 | 21.37 | 0.00 |
| 0.1 | 0.01 | -2.69 | 1.00 | 23.06 | 0.00 |
| 0.2 | 0.02 | -2.53 | 1.00 | 24.74 | 0.01 |
| 0.3 | 0.02 | -2.36 | 1.00 | 26.43 | 0.01 |
| 0.4 | 0.02 | -2.19 | 1.00 | 28.12 | 0.01 |
| 0.5 | 0.04 | -2.02 | 1.00 | 29.80 | 0.02 |
| 0.6 | 0.04 | -1.85 | 1.30 | 31.49 | 0.03 |
| 0.7 | 0.06 | -1.68 | 1.64 | 33.18 | 0.05 |
| 0.8 | 0.06 | -1.51 | 1.97 | 34.86 | 0.07 |
| 0.9 | 0.09 | -1.34 | 2.31 | 36.55 | 0.09 |
| 1.0 | 0.15 | -1.18 | 2.65 | 38.24 | 0.12 |
| 1.1 | 0.15 | -1.01 | 2.98 | 39.92 | 0.16 |
| 1.2 | 0.21 | -0.84 | 3.32 | 41.61 | 0.20 |
| 1.3 | 0.21 | -0.67 | 3.66 | 43.30 | 0.25 |
| 1.4 | 0.29 | -0.50 | 4.00 | 44.99 | 0.31 |
| 1.5 | 0.40 | -0.33 | 4.33 | 46.67 | 0.37 |
| 1.6 | 0.40 | -0.16 | 4.67 | 48.36 | 0.43 |
| 1.7 | 0.52 | 0.00 | 5.01 | 50.05 | 0.50 |
| 1.8 | 0.52 | 0.17 | 5.35 | 51.73 | 0.57 |
| 1.9 | 0.64 | 0.34 | 5.68 | 53.42 | 0.63 |
| 2.0 | 0.77 | 0.51 | 6.02 | 55.11 | 0.70 |
| 2.1 | 0.77 | 0.68 | 6.36 | 56.79 | 0.75 |
| 2.2 | 0.84 | 0.85 | 6.70 | 58.48 | 0.80 |
| 2.3 | 0.84 | 1.02 | 7.03 | 60.17 | 0.85 |
| 2.4 | 0.89 | 1.19 | 7.37 | 61.85 | 0.88 |
| 2.5 | 0.93 | 1.35 | 7.71 | 63.54 | 0.91 |
| 2.6 | 0.93 | 1.52 | 8.05 | 65.23 | 0.94 |
| 2.7 | 0.96 | 1.69 | 8.38 | 66.91 | 0.95 |
| 2.8 | 0.96 | 1.86 | 8.72 | 68.60 | 0.97 |
| 2.9 | 0.98 | 2.03 | 9.00 | 70.29 | 0.98 |
| 3.0 | 1.00 | 2.20 | 9.00 | 71.97 | 0.99 |

Table S8 – Norm values for the FMI-13R, *Acceptance* factor

| **Mean** | **Percent (empirical)** | **z** | **Stanine** | **T** | **Percent (normal)** |
| --- | --- | --- | --- | --- | --- |
| 0.0 | 0.01 | -2.99 | 1.00 | 20.06 | 0.00 |
| 0.1 | 0.01 | -2.82 | 1.00 | 21.78 | 0.00 |
| 0.2 | 0.01 | -2.65 | 1.00 | 23.51 | 0.00 |
| 0.3 | 0.02 | -2.48 | 1.00 | 25.24 | 0.01 |
| 0.4 | 0.02 | -2.30 | 1.00 | 26.96 | 0.01 |
| 0.5 | 0.03 | -2.13 | 1.00 | 28.69 | 0.02 |
| 0.6 | 0.04 | -1.96 | 1.08 | 30.42 | 0.03 |
| 0.7 | 0.04 | -1.79 | 1.43 | 32.14 | 0.04 |
| 0.8 | 0.05 | -1.61 | 1.77 | 33.87 | 0.05 |
| 0.9 | 0.08 | -1.44 | 2.12 | 35.60 | 0.07 |
| 1.0 | 0.13 | -1.27 | 2.47 | 37.33 | 0.10 |
| 1.1 | 0.13 | -1.09 | 2.81 | 39.05 | 0.14 |
| 1.2 | 0.17 | -0.92 | 3.16 | 40.78 | 0.18 |
| 1.3 | 0.24 | -0.75 | 3.50 | 42.51 | 0.23 |
| 1.4 | 0.24 | -0.58 | 3.85 | 44.23 | 0.28 |
| 1.5 | 0.33 | -0.40 | 4.19 | 45.96 | 0.34 |
| 1.6 | 0.42 | -0.23 | 4.54 | 47.69 | 0.41 |
| 1.7 | 0.42 | -0.06 | 4.88 | 49.41 | 0.48 |
| 1.8 | 0.51 | 0.11 | 5.23 | 51.14 | 0.55 |
| 1.9 | 0.63 | 0.29 | 5.57 | 52.87 | 0.61 |
| 2.0 | 0.75 | 0.46 | 5.92 | 54.60 | 0.68 |
| 2.1 | 0.75 | 0.63 | 6.26 | 56.32 | 0.74 |
| 2.2 | 0.82 | 0.80 | 6.61 | 58.05 | 0.79 |
| 2.3 | 0.87 | 0.98 | 6.96 | 59.78 | 0.84 |
| 2.4 | 0.87 | 1.15 | 7.30 | 61.50 | 0.87 |
| 2.5 | 0.90 | 1.32 | 7.65 | 63.23 | 0.91 |
| 2.6 | 0.94 | 1.50 | 7.99 | 64.96 | 0.93 |
| 2.7 | 0.94 | 1.67 | 8.34 | 66.68 | 0.95 |
| 2.8 | 0.96 | 1.84 | 8.68 | 68.41 | 0.97 |
| 2.9 | 0.98 | 2.01 | 9.00 | 70.14 | 0.98 |
| 3.0 | 1.00 | 2.19 | 9.00 | 71.87 | 0.99 |

Table S9 – Mapping of Stanine values to mean value, FMI-13R, *Presence* factor

| **Stanine** | **Lower Bound Mean Score** | **Upper Bound Mean Score** |
| --- | --- | --- |
| 1 | -Inf | 0.7 |
| 2 | 0.7 | 1.0 |
| 3 | 1.0 | 1.3 |
| 4 | 1.3 | 1.5 |
| 5 | 1.5 | 1.8 |
| 6 | 1.8 | 2.1 |
| 7 | 2.1 | 2.4 |
| 8 | 2.4 | 2.7 |
| 9 | 2.7 | Inf |

Table S10 – Mapping of Stanine values to mean value, FMI-13R, *Acceptance* factor

| **Stanine** | **Lower Bound Mean Score** | **Upper Bound Mean Score** |
| --- | --- | --- |
| 1 | -Inf | 0.7 |
| 2 | 0.7 | 1.0 |
| 3 | 1.0 | 1.3 |
| 4 | 1.3 | 1.6 |
| 5 | 1.6 | 1.9 |
| 6 | 1.9 | 2.2 |
| 7 | 2.2 | 2.5 |
| 8 | 2.5 | 2.7 |
| 9 | 2.7 | Inf |

Table S11 – Mapping of Stanine values to mean value, FMI-14, unifactorial solution

| **Stanine** | **Lower Bound Mean Score** | **Upper Bound Mean Score** |
| --- | --- | --- |
| 1 | -Inf | 0.8 |
| 2 | 0.8 | 1.1 |
| 3 | 1.1 | 1.3 |
| 4 | 1.3 | 1.6 |
| 5 | 1.6 | 1.8 |
| 6 | 1.8 | 2.1 |
| 7 | 2.1 | 2.3 |
| 8 | 2.3 | 2.6 |
| 9 | 2.6 | Inf |
